# Protein Cytl1: its role in chondrogenesis, cartilage homeostasis, and disease

**DOI:** 10.1007/s00018-019-03137-x

**Published:** 2019-05-14

**Authors:** Sipin Zhu, Vincent Kuek, Samuel Bennett, Huazi Xu, Vicki Rosen, Jiake Xu

**Affiliations:** 1 Department of Orthopaedics, The Second Affiliated Hospital and Yuying Children’s Hospital of Wenzhou Medical University, Wenzhou, 325000 Zhejiang China; 2 Molecular Laboratory, Division of Regenerative Medicine, School of Biomedical Sciences, University of Western Australia, Perth, WA 6009 Australia; 3 Developmental Biology, Harvard School of Dental Medicine, Boston, MA 02115 USA

**Keywords:** Birth, Heart, Immune, Toxic, Genetic

## Abstract

Cytokine-like protein 1 (Cytl1), also named Protein C17 or C4orf4 is located on human chromosome 4p15-p16 and encodes a polypeptide of 126 amino acid residues that displays characteristics of a secretory protein. Cytl1 is expressed by a sub-population of CD34^+^ human mononuclear cells from bone marrow and cord blood, and by chondrocytes (cartilage-forming cells). In this review, we explore evidence suggesting that Cytl1 may be involved in the regulation of chondrogenesis, cartilage homeostasis and osteoarthritis progression, accompanied by the modulation of Sox9 and insulin-like growth factor 1 expression. In addition, Cytl1 exhibits chemotactic and pro-angiogenic biological effects. Interestingly, CCR2 (C–C chemokine receptor type 2) has been identified as a likely receptor for Cytl1, which mediates the ERK signalling pathway. Cytl1 also appears to mediate the TGF-beta-Smad signalling pathway, which is hypothetically independent of the CCR2 receptor. More recently, studies have also potentially linked Cytl1 with a variety of conditions including cardiac fibrosis, smoking, alcohol dependence risk, and tumours such as benign prostatic hypertrophy, lung squamous cell carcinoma, neuroblastoma and familial colorectal cancer. Defining the molecular structure of Cytl1 and its role in disease pathogenesis will help us to design therapeutic approaches for Cytl1-associated pathological conditions.

## Introduction

Cytokine-like protein 1 (Cytl1) was initially discovered in relation to a rare population of human bone marrow (BM) and cord blood (CB) mononuclear cells that function as haematopoietic stem/progenitor cells and bear the CD34 surface marker (CD34^+^) [1]. The Cytl1 gene, also called the C17 gene, is not expressed in mature haematopoietic cells that lack CD34 expression, and was identified to elucidate possible molecular mechanisms regulating the maturation of haematopoietic progenitor cells [1]. Although the Cytl1 gene has been mapped to human chromosome 4p15–p16, the definitive molecular structure of the Cytl1 protein remains to be determined, and there is uncertainty as to the true classification of Cytl1 due to its cytokine-like properties and chemokine activity.

Evidence indicates that Cytl1 is a secretory protein with a predicted structure characteristic of haematopoietic cytokines and interleukins, and capable of performing immunobiological functions [1]. The cellular signalling mechanisms and potential receptors for Cytl1 remain largely unknown. Cytl1 appears to play a role in chondrogenesis, cartilage homeostasis, and osteoarthritis progression. Recent research has found that the Cytl1 gene may increase individual susceptibility to a number of systemic diseases, and function in an immunomodulatory capacity with implications for toxicology relating to early childhood illness. Further evidence suggests that Cytl1 may have an emerging role in tissue regeneration and as a target for the delivery of therapeutic agents for disease control.

## Molecular structure, gene expression, and function of Cytl1

Cytl1, also called Protein C17, or C4orf4 is located on human chromosome 4p15–p16 [1]. It has an amino acid signal peptide from residues aa 1 to aa 22, and characteristics of a secreted protein. Cloned Cytl1 cDNA encodes a polypeptide of 136 amino acids, and the predicted molecular mass of Cytl1 is estimated to be 15.6 kDa [1]. Cytl1 is specifically expressed in a rare population of human bone marrow and cord blood mononuclear cells that bear CD34 positivity and function as haematopoietic stem/progenitor cells [1].

**Fig. 1:**
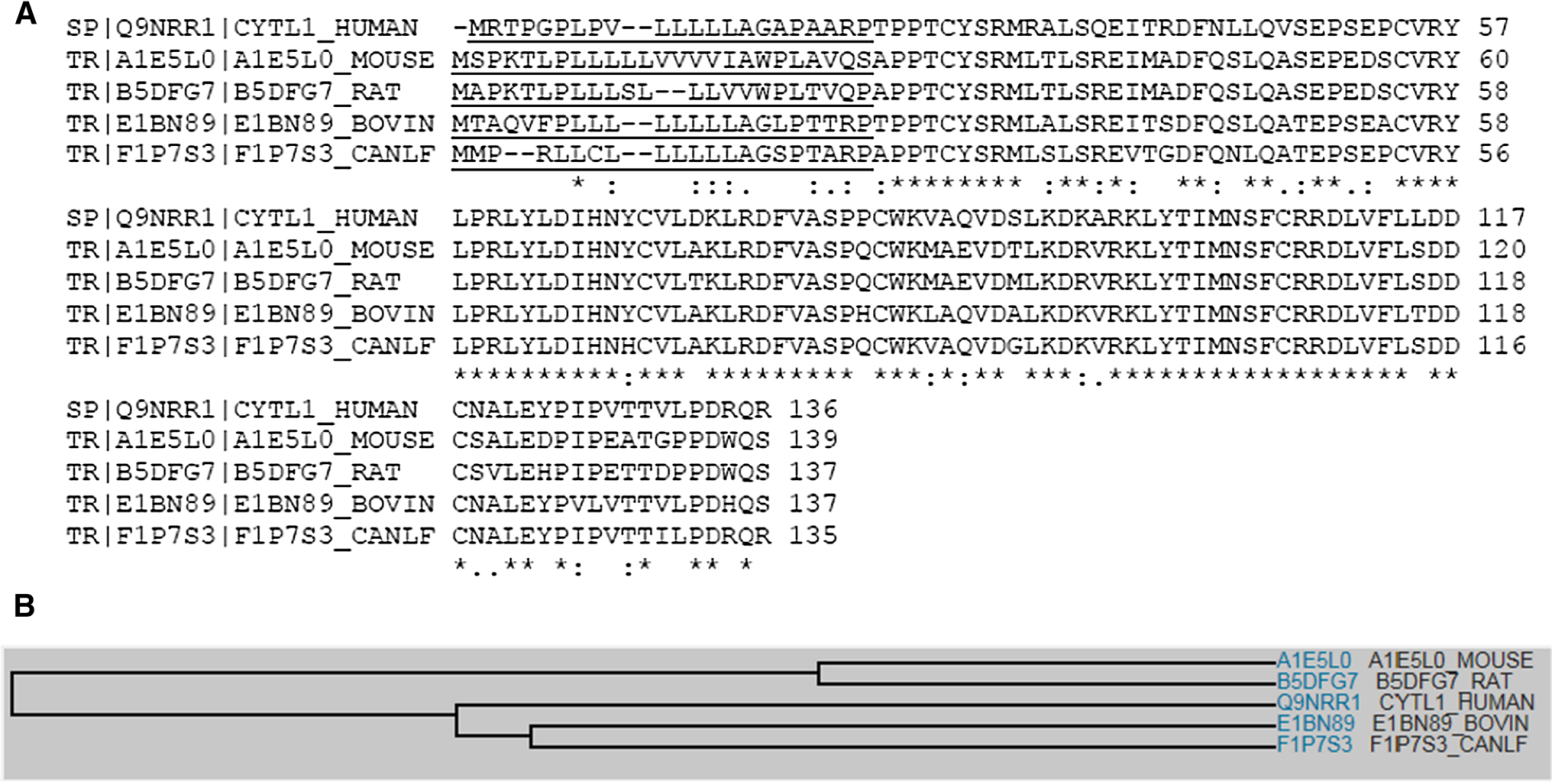
**a** Multiple sequence alignment showing substantial sequence homology among Cytl1 amino acid sequences in human, rat and mouse. **b** Family tree analyses among Cytl1 amino acid sequences in human, rat and mouse

**Fig. 2:**
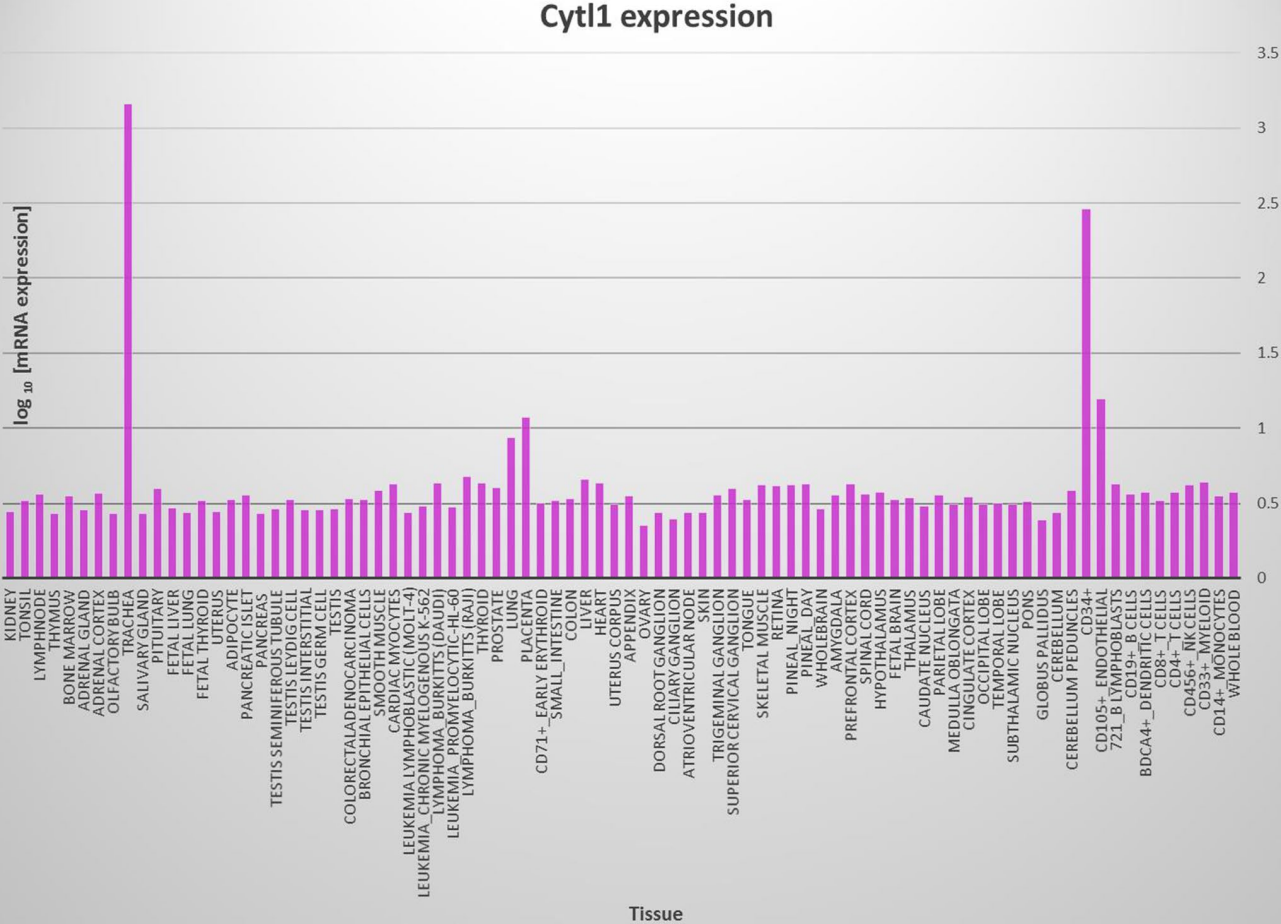
BioGPS analyses showing the expression profiling of human gene among various tissues and cells. Note that Cytl1 is most specifically expressed in trachea, a cartilage-rich tissue, and in CD34+ cells

Multiple sequence alignment analysis reveals that there are substantial sequence similarities among human, mouse, rat, bovine and dog homologs of Cytl1 (Fig. 1a). Family tree analysis shows that human Cytl1 is most closely related to bovine and dog followed by mouse and rat (Fig. 1b). Gene profiling analysis by BioGPS reveals that the human Cytl1 gene is most abundantly and specifically expressed in trachea, a cartilage-rich tissue, and CD34-positive cells (Fig. 2).

**Fig. 3:**
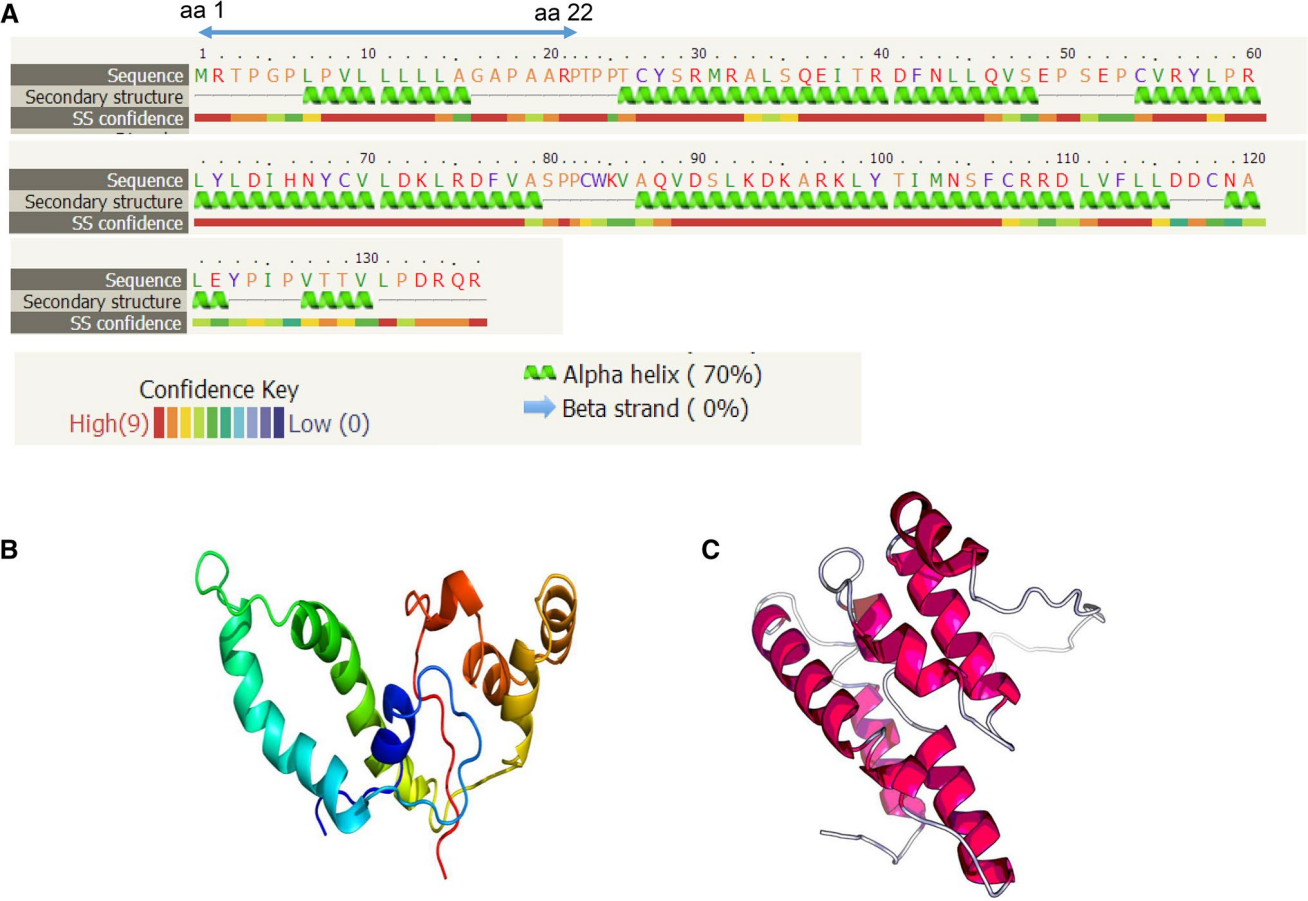
Molecular structure of Cytl1. **a** Secondary structure of Cytl1 showing that it contains a signalling sequence from amino acid residues aa 1 to aa 22, and mainly three large alpha helix domains and two small motifs with no predicted beta strand according to the web-link-based bioinformatics analysis (http://twitter.com/phyre2server). **b** Tertiary structure predicted by RaptorX template-based protein structure modelling according to the web-link based bioinformatics analysis (http://raptorx.uchicago.edu/StructurePrediction). **c** Tertiary structure predicted by the Phyre2 web portal for protein modelling based on the web-link bioinformatics analysis (http://twitter.com/phyre2server)

Analysis of the molecular structure of Cytl1 revealed that it contains a signal peptide at its N terminus from amino acid residues aa 1 to aa 22, and three large alpha helix domains and two small motifs with no predicted beta strand. Phyre2 and Raptor X are community-wide web-based resources for bioinformatics analyses and prediction of 3D protein structure and function [2–4]. Utilizing Phyre2 and Raptor X, the tertiary structure of Cytl1 was predicted to have characteristics of alpha helix domain folding (Fig. 3b, c) [2–4].

**Fig. 4:**
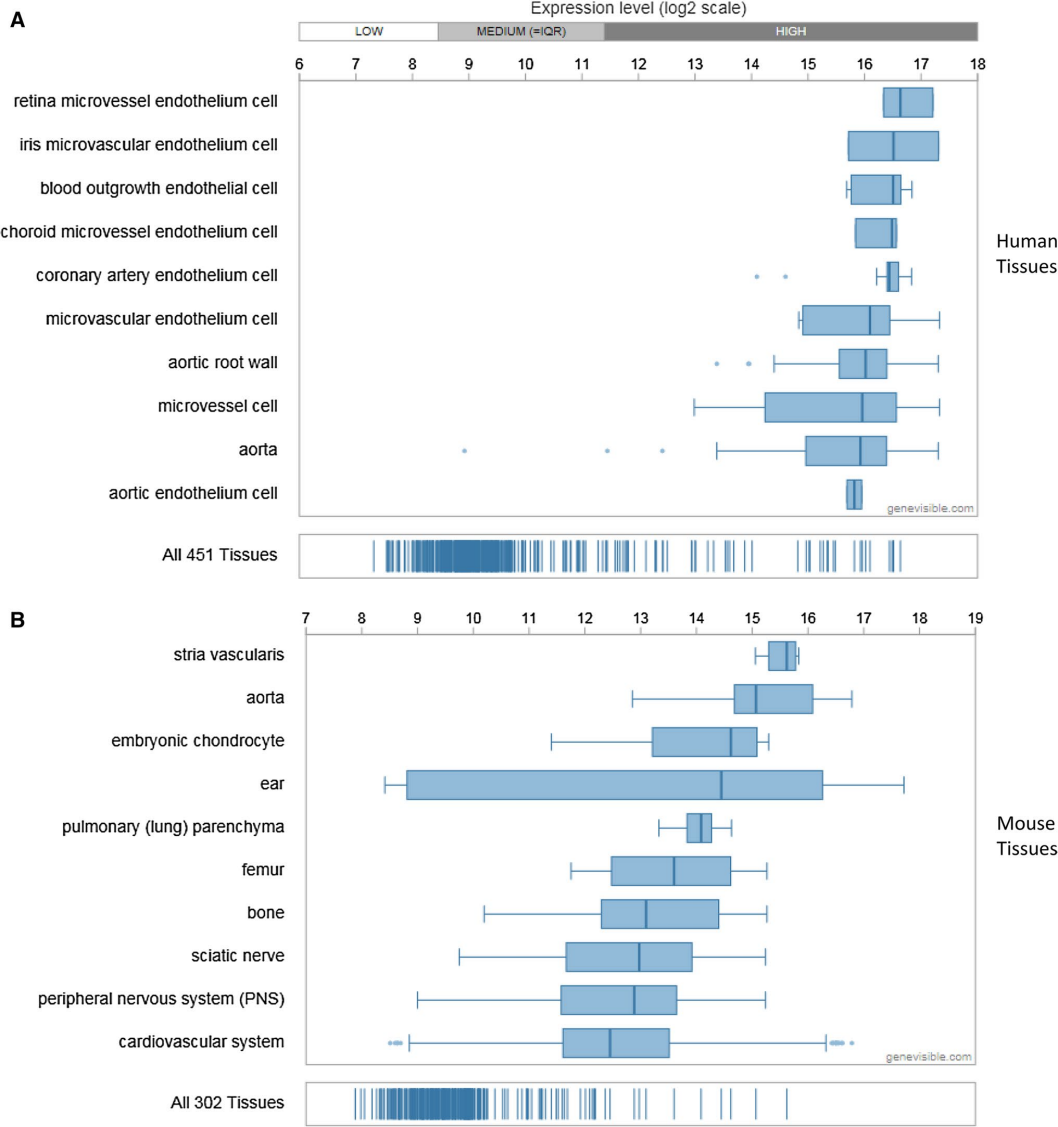
Expression analyses performed by Genevisible (http://genevisible.com) showing the Cytl1 expression in both human and mouse tissues, including the 10 most highly expressed tissues for each species

**Fig. 5:**
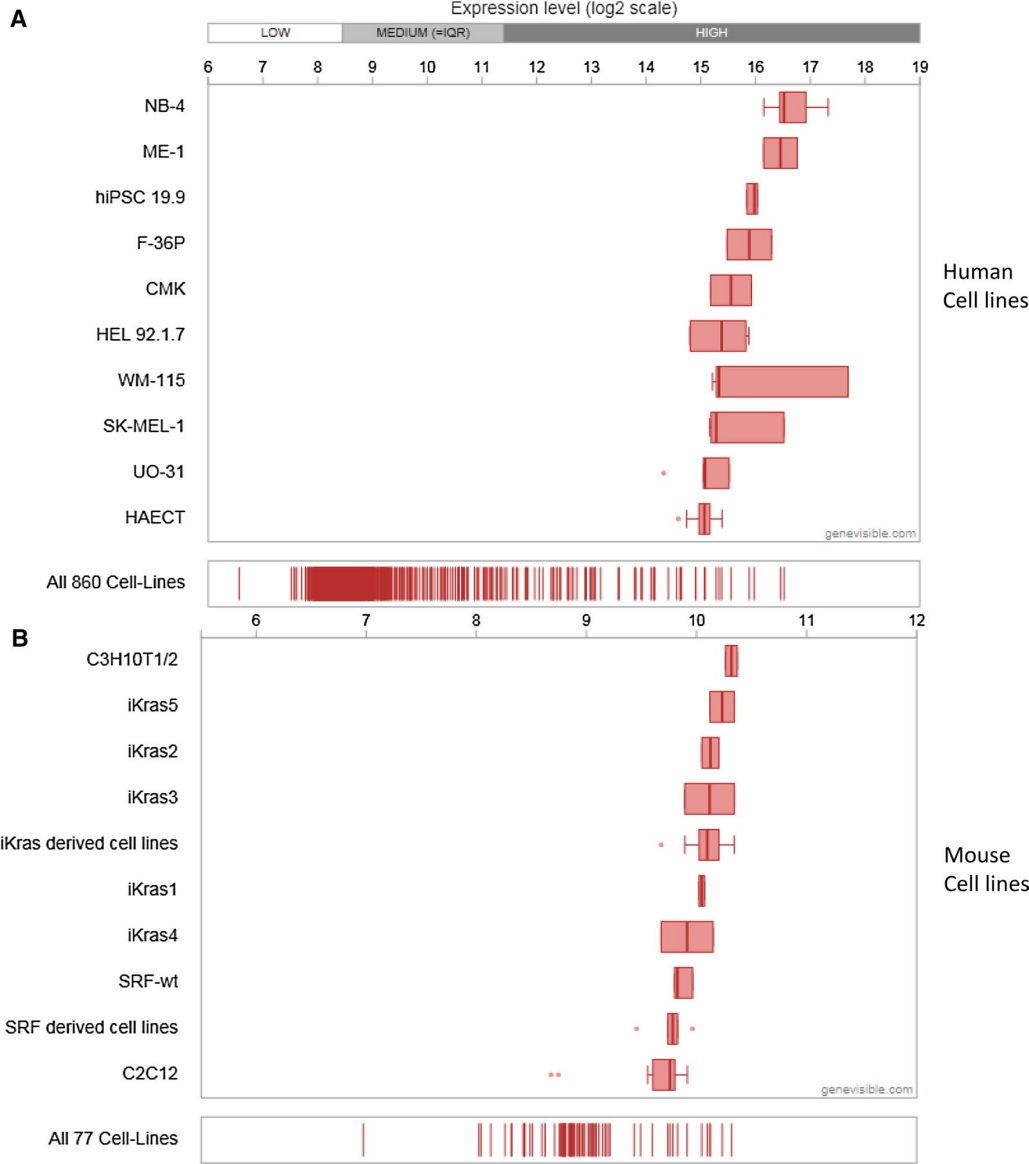
Expression analyses performed by Genevisible^®^http://genevisible.com showing the Cytl1 expression in both human and mouse cell lines, including the 10 most highly expressed cell lines for each species

Extensive bioinformatics analysis using Genevisible^®^ indicates that Cytl1 is also expressed in human and mouse tissues (Fig. 4), with Cytl1 most abundantly expressed in retina micro-vessel endothelium cells, iris microvascular endothelium cells, and blood outgrowth endothelial cells in humans, as well as stria vascularis, aorta, and embryonic chondrocytes in mouse tissues [5] (Fig. 4). Further research is necessary to investigate the expression of CD34 by these tissues. In addition, extensive bioinformatics analysis using Genevisible^®^ indicates that Cytl1 is expressed in human and mouse cell lines (Fig. 5), with Cytl1 most abundantly expressed in the NB-4, ME-1 and hiPSC19.9 human cell lines, as well as the C3H10T1/2, iKras5, and iKras2 cell lines (Fig. 5) [5].

## Cytl1 receptor, signalling and transcriptional activation

**Fig. 6:**
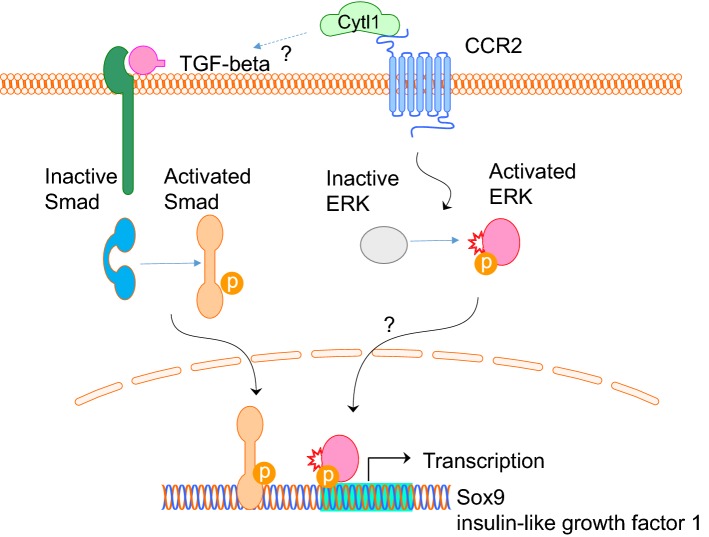
Putative signalling pathway of Ctyl1. Cytl1 appears to act on the CCR2 receptor to activate ERK signalling and downstream transcriptional activation of Sox9 and insulin-like growth factor 1. Alternatively, Cytl1 might also act via the TGF-beta-Smad signalling pathway, independent of CCR2, to activate downstream transcriptional activation of Sox9 and insulin-like growth factor 1

Cytl1 was found to be predominantly expressed in chondrocytes and cartilage [6, 7]. Exogenous addition of Cytl1 induced mesenchymal cells to undergo chondrogenic differentiation by micro-mass culture, but did not affect the hypertrophic maturation of chondrocytes [7]. The chondrogenic effect of Cytl1 is exerted by the induction of Sox9 transcriptional activity and the expression of insulin-like growth factor 1 [7] (Fig. 6).

Structurally, Cytl1 appears to resemble an IL8-like chemokine folding, with particular similarity to monocyte chemoattractant protein 1, MCP-1 (or CCL2), and appears to have a functional chemokine receptor, CCR2, that is likely to be involved in the pathogenesis of osteoarthritis and rheumatoid arthritis [8]. Recent research has shown that Cytl1 exhibits a chemotactic effect on monocytes and macrophages via its receptor CCR2B-ERK signalling pathway [9] (Fig. 6). This direct signalling effect is further confirmed because macrophages from wild-type mice but not from CCR2 null mice are chemotactically responsive to recombinant Cytl1 proteins [9].

Interestingly, Cytl1, also appears to be involved in the pathogenesis of cardiac fibrosis (CF) and heart failure (HF), and may be structurally and functionally related to MCP-1 (or CCL2) [10]. Cytl1 has been shown to induce the expression of TGF-beta2, and appears to play an essential role in CF and HF via activation of the TGF-beta-Smad signalling pathway [10]. Further, the pro-fibrotic activity of Cytl1 was unaffected by antagonization of the CCR2 receptor, which indicates that Cytl1 may have more than two receptors, and that the receptor that is responsible for mediating the pro-fibrotic activity of Cytl1 in the heart remains to be identified [10] (Fig. 6). In addition, Cytl1 appears to be produced by endothelial progenitor cells, particularly endothelial colony-forming cells (ECFCs), and was found to have pro-angiogenic effects by inducing sprouting and vessel formation that is comparable to VEGF-A [11]. O-glycosylation on two neighbouring threonines in the C-terminus appears to be important for the pro-angiogenic bioactivity of Cytl1 [11]. It is suggested that this angiogenic function is largely independent of VEGF-A [11]. Thus further studies are required to demonstrate the detailed mechanisms by which Cytl1 may mediate pro-angiogenic functions and may be implicated in the pathogenesis of cardiac fibrosis and heart failure.

## The role of Cytl1 in cartilage homeostasis and osteoarthritis development

Osteoarthritis (OA) is a chronic debilitating joint disease characterized by the degradation of articular cartilage, subchondral bone sclerosis, osteophyte formation, and inflammation of the synovial membrane [12]. Further, research implicates invasive angiogenesis, the critical role of pro-inflammatory secretory molecules, and chondrocyte dedifferentiation in the pathophysiology of OA [13–15]. Cytl1 is known to regulate the chondrogenic differentiation of mesenchymal cells and increase the expression of interleukin-1 (IL-1) during chondrogenesis [7]. Additionally, Cytl1 appears to have strong pro-angiogenic effects, comparable to vascular endothelial growth factor-A (VEGF-A), has chemotactic activity towards monocytes and macrophages via the CCR2B receptor, and its expression appears to be downregulated during OA progression [9, 11, 16]. Taken together, these findings suggest that Cytl1 is implicated in the progression of OA and could be a potential therapeutic target for intervention.

Although Cytl1 was initially found to be expressed by a human population of CD34^+^ bone marrow and cord blood cells, Cytl1 was more recently reported to be expressed by chondrocytes, which is suggestive of a role in chondrogenesis and cartilage homeostasis [1, 17, 18]. However, Cytl1 gene knockout did not affect chondrogenesis or cartilage development as Cytl1 (−/−) mice exhibited normal endochondral ossification and long bone development with no abnormal ultrastructural features of articular cartilage, including matrix organization and chondrocyte morphology [6]. Notably, Cytl1 (−/−) mice showed more cartilage destruction after the medial meniscus of mouse knee joints was disturbed, as compared with wild-type littermates [6]. Further, Cytl1 was found to be preferentially expressed in chondrocytes and in cartilage-rich tissues, and systemic expression of Cytl1 in vivo inhibits collagen antibody-induced arthritis in mice [18]. More recently, Cytl1, was identified as being differentially expressed in post-traumatic osteoarthritis (PTOA), indicative that Cytl1 may be a potential target for specific intervention in early PTOA, and further suggesting the role of Cytl1 in osteoarthritis development [16]. Also, the expression levels of Cytl1 were decreased in OA cartilage of humans and experimental mice [6]. Collectively, multiple lines of evidence suggest that, rather than regulating physiological cartilage and bone development, Cytl1 is involved mainly in the maintenance of cartilage homeostasis, and that loss of Cytl1 function is associated with osteoarthritis progression [6, 7, 18, 19].

In addition to its role in cartilage homeostasis and osteoarthritis progression, Cytl1 has been found to be involved in the regulation of macrophage migration [8, 9, 20]. Structural–functional analyses of Cytl1 suggests that it is likely to adopt an IL8-like chemokine fold, in particular similar to CCL2 (monocyte chemoattractant protein 1, MCP-1), and Cytl1 consistently appears to exhibit a chemotactic effect for monocytes and macrophages [8, 9]. Thus, Cytl1 appears to have cytokine-like properties combined with chemokine abilities, and, therefore, definitive determination of the tertiary structure of Cytl1 is required to make a true classification [20]. Current evidence indicates that Cytl1 may be an important atypical cytokine-like protein ligand with functional chemokine receptor, CCR2 [20].

## The potential role of Cytl1 in cancers and disease pathogenesis

Cytl1 is associated with several types of cancers [21–24]. It was identified as one of the three candidate genes that harboured rare loss-of-function variants in both early-onset and familial colorectal cancer (CRC) in a study analysing in 3374 Finnish and 58,112 non-Finnish controls [21]. Using a combination of the whole-genome DNA methylation pattern and the gene-expression profile, Cytl1 was also identified as one of the candidate genes that is regulated by DNA methylation in human lung squamous cell carcinoma [22]. In addition, Cytl1 gene expression was detected in human tumour cell lines, particularly SH-SY5Y, and human neuroblastoma (NB) tissues, and decreased expression of the Cytl1 gene when blocked by siRNA results in reduced cell proliferation, migration and invasion activities by SH-SY5Y cells [24]. These findings are indicative of an association of Cytl1 expression with growth and metastasis of neuroblastoma cells, and suggest that Cytl1 may be a potential therapeutic target and diagnosis biomarker for NB [24]. Further, previous studies have revealed that Cytl1 expression together with secreted CXC-type chemokinesis was associated with benign prostatic hypertrophy [23].

A pro-angiogenic function of Cytl1 was identified in endothelial progenitors, suggestive of its role in vessel formation and thus its applicable potential for tissue regeneration in ischemic pathologies [11]. In addition, Cytl1 was also found expressed in the endometrium as an ovarian hormone-dependent gene, and regulates the proliferation of HEC-1-A and RL95-2 cells, suggesting a putative role of Cytl1 in the regulation of embryo implantation during early pregnancy [17]. Further, differential expression of Cytl1, together with Efnb1, and Tex26, was found in trophectoderm biopsies, which suggests that Cytl1 might be associated with successful embryo implantation and live birth [25].

Alcohol dependence (AD) is a complex disease, with genetic and environmental aetiological factors, resulting in uncontrolled alcohol consumption. A recent genome-wide association study (GWAS) found that certain haplotypes within the Cytl1 gene were significantly associated with an increased risk of alcohol dependence (AD), suggesting that the Cytl1 gene may play an important role in the susceptibility to AD [26]. The possible underlying molecular and cellular mechanisms of this association are unknown and further studies are required to elucidate the significance of Cytl1 gene variants in relation to AD.

Tobacco smoking is a risk factor for diseases including cancer, cardiovascular disease, and diabetes. A recent twin study investigating DNA methylation and gene expression markers in tissues outside the lung, found that Cytl1 is one of five genes, including Ahrr, Cyp1a1, Cyp1b1, and F2rl3, that appear to be hypomethylated and upregulated in smokers, indicating that DNA methylation and expression levels of Cytl1 may serve as an associated marker of smoking-associated impaired metabolic health [27].

Perfluoroalkyl and polyfluoroalkyl substances (PFAS) are synthetic fluorinated compounds that have been produced in large-scale quantities for consumer-products and industrial use, and are considered environmental pollutants with toxic properties [28]. Prenatal human exposure to PFAS occurs via placental transfer and is associated with postnatal and early childhood immunosuppression [28]. Cytl1 is one of several immunomodulatory genes that were also found to be PFAS exposure-associated genes that are associated with early childhood immunosuppression and increased susceptibility to common colds [28]. Therefore, Cytl1 appears to be one of several immunomodulatory genes that might serve as a PFAS toxicogenomics marker [28].

Consistent with the viewpoint that Cytl1 has immune-regulatory properties: Cytl1 bears sequence homology to IL-2, may be a member of the IL-2 cytokine family, and appears to contribute to immune homeostasis systemically, and in a tissue-specific capacity [18]. Further investigation of the immunobiological functions, potential receptors, and signalling pathways of Cytl1 is required for this relatively novel protein [9]. Understanding the mechanisms by which Cytl1 regulates these pathological conditions will be important in designing therapeutic targets for Cytl1.

## Conclusion

Cytokine-like 1 is a secretory protein that was discovered by its expression in a sub-population of CD34^+^ haematopoietic progenitor/stem cells, and appears to be expressed mainly in CD34^+^ cells, chondrocytes, and cartilage-rich tissues [1, 7]. Cytl1 is located on human chromosome 4p15–p16, and has cytokine properties and chemokine activity, supporting the hypothesis that Cytl1 functions via immune-regulation. Bioinformatics analyses indicate that Cytl1 is expressed in human and mouse tissues, notably ocular microvascular endothelium, cardiovascular, and cartilaginous tissue. Cytl1 appears to regulate chondrogenesis via stimulating Sox9 and insulin-like growth factor 1 transcriptional activity [7]. Collective evidence suggests that Cytl1 may induce chondrogenesis, and is required for cartilage homeostasis, and the prevention of osteoarthritis progression, rather than the regulation of cartilage and bone development [6]. Cytl1 appears to have a functional chemokine receptor, CCR2, which may regulate ERK signalling to stimulate Sox9 and insulin-like growth factor 1 transcriptional activity. In addition, Cytl1 also appears to activate the TGF-beta-SMAD signalling pathway via an unidentified receptor, and play an important role in cardiac fibrosis and heart failure. Recent research indicates that Cytl1 may be associated with a number of cancers and disease pathogeneses, including neuroblastoma, alcohol dependence, and smoking-associated impaired metabolic health. Cytl1 may also be associated with successful embryo implantation and live birth. Further research is necessary to explore Cytl1 as a potential target for the delivery of therapeutic agents for tissue regeneration and disease control.

## References

[1] Liu X, Rapp N, Deans R, Cheng L (2000). Molecular cloning and chromosomal mapping of a candidate cytokine gene selectively expressed in human CD34+ cells. Genomics.

[2] Kelley LA, Mezulis S, Yates CM, Wass MN, Sternberg MJE (2015). The Phyre2 web portal for protein modeling, prediction and analysis. Nat Protoc.

[3] Kallberg M, Wang H, Wang S, Peng J, Wang Z, Lu H, Xu J (2012). Template-based protein structure modeling using the RaptorX web server. Nat Protoc.

[4] Kallberg M, Margaryan G, Wang S, Ma J, Xu J (2014). RaptorX server: a resource for template-based protein structure modeling. Methods Mol Biol.

[5] Hruz T, Laule O, Szabo G, Wessendorp F, Bleuler S, Oertle L, Widmayer P, Gruissem W, Zimmermann P (2008). Genevestigator v3: a reference expression database for the meta-analysis of transcriptomes. Adv Bioinform.

[6] Jeon J, Oh H, Lee G, Ryu JH, Rhee J, Kim JH, Chung KH, Song WK, Chun CH, Chun JS (2011). Cytokine-like 1 knock-out mice (Cytl1−/−) show normal cartilage and bone development but exhibit augmented osteoarthritic cartilage destruction. J Biol Chem.

[7] Kim JS, Ryoo ZY, Chun JS (2007). Cytokine-like 1 (Cytl1) regulates the chondrogenesis of mesenchymal cells. J Biol Chem.

[8] Tomczak A, Pisabarro MT (2011). Identification of CCR8-binding features in Cytl1 by a CCL2-like chemokine model. Proteins.

[9] Wang X, Li T, Wang W, Yuan W, Liu H, Cheng Y, Wang P, Zhang Y, Han W (2016). Cytokine-like 1 chemoattracts monocytes/macrophages via CCR9. J Immunol.

[10] Kim J, Kim J, Lee SH, Kepreotis SV, Yoo J, Chun JS, Hajjar RJ, Jeong D, Park WJ (2016). Cytokine-like 1 regulates cardiac fibrosis via modulation of TGF-beta signaling. PLoS One.

[11] Schneller D, Hofer-Warbinek R, Sturtzel C, Lipnik K, Gencelli B, Seltenhammer MH, Wen M, Testori J, Bilban M, Borowski A, Windwarder M, Kapel SS, Besemfelder E, Cejka P, Habertheuer A, Schlechta B, Majdic O, Altmann F, Kocher AA, Augustin HG, Luttmann W, Hofer E (2018). Cytokine-like 1 is a novel pro-angiogenic factor secreted by and mediating functions of endothelial progenitor cells. Circ Res.

[12] Charlier E, Deroyer C, Ciregia F, Malaise O, Neuville S, Plener Z, Malaise M, de Seny D (2019). Chondrocyte dedifferentiation and osteoarthritis (OA). Biochem Pharmacol.

[13] Vadala G, Russo F, Musumeci M, Giacalone A, Papalia R, Denaro V (2018). Targeting VEGF-A in cartilage repair and regeneration: state of the art and perspectives. J Biol Regul Homeost Agents.

[14] Kapoor M, Martel-Pelletier J, Lajeunesse D, Pelletier J-P, Fahmi H (2010). Role of proinflammatory cytokines in the pathophysiology of osteoarthritis. Nat Rev Rheumatol.

[15] Deroyer C, Charlier E, Neuville S, Malaise O, Gillet P, Kurth W, Chariot A, Malaise M, Dominique S (2019). CEMIP (KIAA1199) induces a fibrosis-like process in osteoarthritic chondrocytes. Cell Death Dis.

[16] Sieker JT, Proffen BL, Waller KA, Chin KE, Karamchedu NP, Akelman MR, Perrone GS, Kiapour AM, Konrad J, Murray MM, Fleming BC (2018). Transcriptional profiling of articular cartilage in a porcine model of early post-traumatic osteoarthritis. J Orthop Res.

[17] Ai Z, Jing W, Fang L (2016). Cytokine-like protein 1(Cytl1): a potential molecular mediator in embryo implantation. PLoS One.

[18] Chao C, Joyce-Shaikh B, Grein J, Moshrefi M, Raoufi F, Laface DM, McClanahan TK, Bourne PA, Pierce RH, Gorman DM, Pflanz S (2011). C17 prevents inflammatory arthritis and associated joint destruction in mice. PLoS One.

[19] Stenberg J, Ruetschi U, Skioldebrand E, Karrholm J, Lindahl A (2013). Quantitative proteomics reveals regulatory differences in the chondrocyte secretome from human medial and lateral femoral condyles in osteoarthritic patients. Proteome Sci.

[20] Tomczak A, Singh K, Gittis AG, Lee J, Garboczi DN, Murphy PM (2017). Biochemical and biophysical characterization of cytokine-like protein 1 (CYTL1). Cytokine.

[21] Tanskanen T, Gylfe AE, Katainen R, Taipale M, Renkonen-Sinisalo L, Jarvinen H, Mecklin JP, Bohm J, Kilpivaara O, Pitkanen E, Palin K, Vahteristo P, Tuupanen S, Aaltonen LA (2015). Systematic search for rare variants in Finnish early-onset colorectal cancer patients. Cancer Genet.

[22] Kwon YJ, Lee SJ, Koh JS, Kim SH, Lee HW, Kang MC, Bae JB, Kim YJ, Park JH (2012). Genome-wide analysis of DNA methylation and the gene expression change in lung cancer. J Thorac Oncol.

[23] Begley LA, Kasina S, MacDonald J, Macoska JA (2008). The inflammatory microenvironment of the aging prostate facilitates cellular proliferation and hypertrophy. Cytokine.

[24] Wen M, Wang H, Zhang X, Long J, Lv Z, Kong Q, An Y (2012). Cytokine-like 1 is involved in the growth and metastasis of neuroblastoma cells. Int J Oncol.

[25] Kirkegaard K, Villesen P, Jensen JM, Hindkjaer JJ, Kolvraa S, Ingerslev HJ, Lykke-Hartmann K (2015). Distinct differences in global gene expression profiles in non-implanted blastocysts and blastocysts resulting in live birth. Gene.

[26] Chen XD, Xiong DH, Yang TL, Pei YF, Guo YF, Li J, Yang F, Pan F, Tan LJ, Yan H, Liu XG, Lei SF, Li X, Ning LL, Zhu XZ, Levy S, Kranzler HR, Farrer LA, Gelernter J, Recker RR, Deng HW (2012). ANKRD7 and CYTL1 are novel risk genes for alcohol drinking behavior. Chin Med J (Engl).

[27] Tsai PC, Glastonbury CA, Eliot MN, Bollepalli S, Yet I, Castillo-Fernandez JE, Carnero-Montoro E, Hardiman T, Martin TC, Vickers A, Mangino M, Ward K, Pietilainen KH, Deloukas P, Spector TD, Vinuela A, Loucks EB, Ollikainen M, Kelsey KT, Small KS, Bell JT (2018). Smoking induces coordinated DNA methylation and gene expression changes in adipose tissue with consequences for metabolic health. Clin Epigenetics.

[28] Pennings JL, Jennen DG, Nygaard UC, Namork E, Haug LS, van Loveren H, Granum B (2016). Cord blood gene expression supports that prenatal exposure to perfluoroalkyl substances causes depressed immune functionality in early childhood. J Immunotoxicol.

